# Temporal trends of the in vitro activity of tigecycline and comparator antibiotics against clinical aerobic bacterial isolates collected in Germany, 2006–2014: results of the Tigecycline Evaluation and Surveillance Trial (TEST)

**DOI:** 10.3205/id000025

**Published:** 2016-10-04

**Authors:** Michael Kresken, Barbara Körber-Irrgang, Christian Petrik, Harald Seifert, Arne Rodloff, Karsten Becker

**Affiliations:** 1 Antiinfectives Intelligence GmbH, Rheinbach, Germany; 2 University of Applied Sciences gGmbH, Cologne, Germany; 3 Pfizer Pharma GmbH, Berlin, Germany; 4 University Hospital Cologne, Institute for Medical Microbiology, Immunology and Hygiene, Cologne, Germany; 5 University Hospital Leipzig, Institute for Medical Microbiology and Epidemiology of Infectious Diseases, Leipzig, Germany; 6 University Hospital Münster, Institute of Medical Microbiology, Münster, Germany

**Keywords:** tigecycline, antimicrobial susceptibility, multidrug resistance, Tigecycline Evaluation and Surveillance Trial (TEST), Germany, extended-spectrum beta-lactamase, methicillin-resistant Staphylococcus aureus, vancomycin-resistant enterococci

## Abstract

Given the rapidly changing landscape of antimicrobial resistance, continuous monitoring of antimicrobial susceptibility in clinically relevant bacterial isolates plays an important role in the management of infectious diseases. The Tigecycline Evaluation and Surveillance Trial (TEST) is an ongoing worldwide surveillance programme monitoring the in vitro activity of tigecycline and a panel of representative comparator antibiotics. We report longitudinal susceptibility data on a large set of isolates (n=36,044) from clinically significant bacterial species collected in 25 microbiological laboratories from 2006 to 2014. Trends include a strong increase of carbapenem and levofloxacin resistance in *Acinetobacter* spp., and smaller increasing rates of ESBL-producing *Escherichia coli* and vancomycin-resistant enterococci. Across the reporting period, the tigecycline minimum inhibitory concentrations (MICs) at which 50% and 90% of isolates were inhibited remained stable and susceptibility rates were consistently high (93–100%) for all bacterial species.

## Introduction

As the World Health Organization recently pointed out in its global report, antimicrobial resistance threatens the effective treatment of an increasing range of infectious diseases [1]. Patients with infections caused by multidrug-resistant (MDR) bacteria are generally at elevated risk of unfavorable clinical outcomes and death [2]. In many settings and patient groups, standard antimicrobials are no longer considered adequate choices for empirical therapy of serious infections [3], [4]. A particularly concerning development is the rapid spread of MDR Gram-negative pathogens as infecting and colonizing organisms, which is mostly due to the expansion of genetic determinants associated with extended-spectrum beta-lactamases (ESBL) or carbapenemase production [5].

Tigecycline (Tygacil^®^; Pfizer Inc.) is a glycylcycline antibiotic [6] licensed for complicated intraabdominal infections (cIAI) and complicated skin/soft-tissue infections (cSSTI) since 2006. It exhibits activity against a broad spectrum of aerobic and anaerobic Gram-negative and Gram-positive bacteria including most MDR organsims of the critically important group of “ESCAPE” pathogens (*Enterococcus faecium, Staphylococcus aureus, Clostridium difficile, Acinetobacter baumannii, Pseudomonas aeruginosa*, and Enterobacteriaceae) [7] such as Gram-negative bacteria producing ESBLs and/or carbapenemases, methicillin-resistant *S. aureus* (MRSA) and vancomycin-resistant enterococci (VRE) [8]. 

*P. aeruginosa* is intrinsically resistant to tigecyline. Susceptibility to tigecycline may be reduced in bacteria of the Proteeae tribe, mostly mediated by overexpression of efflux pumps [9]. Acquired resistance has been described in isolates of *A. baumannii*, several Enterobacteriaceae species and Gram-positive cocci [8], [10]. 

In view of the fact that the number of MDR bacterial pathogens has increased over the past 20–30 years, tigecycline plays an important role in the management of complicated bacterial infections. Longitudinal monitoring of the antimicrobial activity of tigecycline is important in assessing the continued usefulness of this agent. As pathogen distribution and resistance patterns show substantial heterogeneity across countries, as evident for Europe from the data compiled by the EARS-Net [11], country-specific data on the tigecycline suceptibility is necessary and has been published for several geographic areas, including Germany [12], [13], [14], [15], [16], [17], [18], [19], [20], [21].

The Tigecycline Evaluation and Surveillance Trial (TEST) monitors the in vitro activity of tigecycline and a panel of representative comparator antibiotics against clinically relevant pathogens from microbiological laboratories worldwide [22]. This report provides susceptibility data of tigecycline and comparator agents collected as part of TEST [23] in Germany between the market introduction of tigecycline in 2006 until 2014, thus expanding earlier German data from TEST reported by Seifert and Dowzicky for the years 2004–2007 [24] to a more recent observation period. 

The present report focussed on (i) bacterial species which are frequently associated with resistance phenotypes and infections in severely ill patients and (ii) antimicrobials frequently used in severe infection and/or representing important groups of antibiotics. A full regularly updated data set of this study is available online via the TEST surveillance website [23].

## Methods

### Bacterial strains

This report includes data from clinical isolates collected by 25 German microbiological laboratories in the time period from 2006 to 2014. Some laboratories, however, did not participate for the entire study period covered in this report. At the beginning of TEST, each participating laboratory had to provide a minimum of 200 isolates per year. These were 135 isolates of Gram-negative pathogens comprising *Acinetobacter* spp. (n=15), *Enterobacter* spp. (n=25), *Escherichia* *coli* (n=25), *Haemophilus* *influenzae* (n=15), *Klebsiella* spp. (*K. oxytoca* and *K. pneumoniae* only; n=25), *P. aeruginosa* (n=20), *Serratia* spp. (n=10) and 65 isolates of Gram-positive pathogens comprising *Enterococcus* spp. (*E. faecium* and *E. faecalis* only; n=15), *S. aureus* (n=25), *Streptococcus agalactiae* (n=10) and *Streptococcus pneumoniae* (n=15). As of 2012, clinical isolates of two additional important genera, *Citrobacter* spp. [25] and *Stenotrophomonas* spp., were included by several laboratories.

Eligible sources of clinical isolates included all sampled body sites as well as medical devices (e.g. catheters, prostheses). Isolates derived from the urinary tract were limited to 25% of the total number. Isolates (one per patient) were collected consecutively from patients with community-acquired or healthcare-associated infections. No restrictions were applied regarding patient age, gender, medical history or previous use of antimicrobials.

### Antimicrobial susceptibility testing

Minimum inhibitory concentrations (MICs) were determined at the participating local laboratories, but partly also at the central laboratory (International Health Management Associates, Inc. [IHMA, Schaumburg, IL, USA]) using broth microdilution as described by the Clinical and Laboratory Standards Institute (CLSI) [26]. Test plates were Sensititre^®^ plates (TREK Diagnostic Systems, West Sussex, England (2008–2011) or MicroScan^®^ panels (Siemens, Sacramento, CA, USA; 2006–2007 and 2012–2014). Test media were prepared fresh on the day of use.

The panel of tested antimicrobials included amoxicillin-clavulanate (AMX/CLV), ampicillin (AMP), ceftriaxone (CXO), levofloxacin (LVX), meropenem (MEM), minocycline (MIN), piperacillin-tazobactam (PTX) and tigecycline (TGC). Gram-negative isolates were also tested for susceptibility to amikacin (AMI), cefepime (CFP) and ceftazidime (CFM), whereas Gram-positive isolates were additionally tested for susceptibility to linezolid (LZD), penicillin (PEN), and vancomycin (VAN). *S*. *pneumoniae* isolates were additionally tested for susceptibility to azithromycin (AZI), clarithromycin (CLA), erythromycin (ERY) and clindamycin (CLI) at the central laboratory. In 2006, imipenem was tested instead of meropenem against the majority of collected isolates.

Confirmation of ESBL production in *E. coli* and *Klebsiella* spp. isolates was performed according to CLSI guidelines using discs of cefotaxime (30 µg), cefotaxime-clavulanic acid (30/10 µg), ceftazidime (30 µg), and ceftazidime-clavulanic acid (30/10 µg) [26]. Antibiotic discs were manufactured by Oxoid, Inc. (Ogdensburg, NY, USA). Mueller-Hinton agar was produced by Remel, Inc. (Lenexa, KS, USA). 

All clinical isolates were sent to the central laboratory which organised the transport of the isolates and managed the study database. It also re-identified the isolates and verified the susceptibility results of 10–15% of the isolates annually.

Quality control (QC) strains included *S. aureus* ATCC 29213, *S. pneumoniae* ATCC 49619, *E. faecalis* ATCC 29212, *E. coli* ATCC 25922, *P. aeruginosa* ATCC 27853, *H.* *influenzae* ATCC 49247, and *H. influenzae* ATCC 49766. MIC data of the clinical isolates were only considered for evaluation if the MICs of the QC strains determined on the day of susceptibility testing were within the quality control ranges defined by CLSI [26]. QC strains used for quality control of ceftazidime and cefotaxime discs were *K. pneumoniae* ATCC 700603 (ESBL-positive) and *E. coli* ATCC 25922 (ESBL-negative), as well as *P. aeruginosa* (ATCC 27853). All isolate data were subject to a quality assurance programme to ensure the validity of the results.

European Committee on Antimicrobial Susceptibility Testing (EUCAST) clinical breakpoints (version 5.0) were applied to antimicrobial agents and organisms for interpretation [27]. Neither EUCAST nor CLSI have set breakpoints for tigecycline against *A. baumannii*. Therefore, the breakpoints proposed by Jones et al. [28] (susceptible: ≤2 µg/mL; resistant: ≥8 µg/mL) were tentatively used for the determination of resistance rates when testing this organism/antibiotic combination. 

## Results

Between 2006 and 2014, the 25 participating laboratories collected a total of 36,044 isolates comprising 12,542 Gram-positive isolates and 23,502 Gram-negative organisms. Annual MIC_50_ and MIC_90_ values as well as resistance rates of tigecycline and comparators for the most clinically relevant species are shown in Table 1 (Tab. 1). A total of 30.1% of these isolates were collected from intensive care patients. 

### Gram-negative pathogens

#### Acinetobacter spp.

The percentage of meropenem-resistant strains among all *Acinetobacter* spp. isolates (n=893) increased from 4,2% in 2007 to 33% in 2014. Resistance to amikacin remained relatively stable at about 10% until 2012, but then increased to more than 30% in 2013/2014. A similar trend was observed for levofloxacin with resistance rates increasing from around 20% to ca. 40% in the last two years. MIC_50_ and MIC_90_ values of tigecycline were in the range of 0.12–0.25 µg/ml and 0.5–2 µg/ml, respectively. Applying the tentative breakpoints proposed by Jones et al. [28], none of the isolates tested were classified as tigecycline-resistant.

#### Escherichia coli

**Figure 1:**
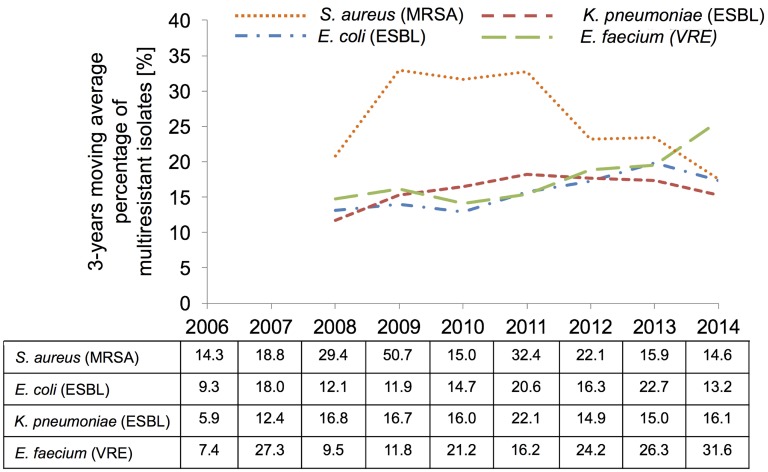
Percentages (%) of multiresistant isolates per total number of isolates for major pathogen species by year of isolation. Annual rates (table) and 3-years moving averages (diagram) are shown.

Among the 2,385 isolates, 16.7% (n=399) showed an ESBL-phenotype. The ESBL rate slightly increased during the test period, with annual rates ranging between 9.3% and 22.7% (Figure 1 (Fig. 1)). Resistance to ceftriaxone and levofloxacin was lowest in the first year of the study period and then varied between 15–25% and 27–42%, respectively, but a clear trend was not observed for either drug. Throughout the study, levofloxacin resistance was more common among ESBL-positive isolates (58–87%) than among ESBL-negative isolates (18–36%), as expected. Piperacillin-tazobactam resistance remained stable, with rates of <10% (data not shown) for ESBL-negative isolates and rates varying between 9% and 38% for ESBL-positive isolates. Meropenem susceptibility was high with no resistant isolates until 2012 and <1% in the last two years. Tigecycline was constantly active against *E. coli* during the entire study period, with MIC_90_ values of 0.25–1 µg/ml and an overall susceptibility rate of 100%.

#### Enterobacter spp.

Meropenem activity against *E. aerogenes* (n=395) and *E. cloacae* (n=1,762) remained very high throughout the reporting period, with resistance rates ranging between 0% and 1.6% for both species. Low resistance rates were also observed for tigecycline (0–7% and 2–7%, respectively). Piperacillin-tazobactam was less active against either species, with resistance rates fluctuating around 20%.

#### Klebsiella spp.

The ESBL rates found for *K. pneumoniae* (n=1,481) ranged between 5.9% to 22.1%, with a mean of 15% (Figure 1 (Fig. 1)). Levofloxacin and piperacillin-tazobactam showed poor activity against ESBL-positive isolates, with resistance rates of 50% and 29% at the end of the study period. In contrast, meropenem remained highly active against *K.* *pneumoniae*, though resistance was observed in 2008 and between 2011 and 2013, albeit at fairly low rates. The MIC_90_ of tigecycline remained at ≤2 µg/ml throughout the reporting period (≤6% resistant isolates overall). However, six out of 38 ESBL-producing isolates exhibited resistance to tigecycline in 2014.

As to *K. oxytoca*, there were 20 ESBL-positive strains among 829 isolates (2.4%). Resistance to meropenem was not detected throughout the reporting period. Tigecycline resistance rates were 0–4% even for ESBL-positive isolates (data not shown). Resistance rates for piperacillin-tazobactam and levofloxacin greatly varied during the study period, with approximately 20% and <10% resistant isolates, respectively, in the last year.

#### Pseudomonas aeruginosa

As expected, tigecycline showed low activity against *P. aeruginosa* (n=1,884). Resistance to ceftazidime was mostly >15% until 2011 and afterwards <15%. No clear trends of susceptibility rates were observed for other antimicrobials, with moderately high rates of resistant isolates recorded for levofloxacin (range 19.8–33.0%), meropenem (6.9–19.4%), and piperacillin-tazobactam (11.0–28.4%).

#### Stenotrophomonas maltophilia

*S. maltophilia* isolates (n=303) were collected in the last 3 years of the reporting period. MIC_50_ and MIC_90_ values recorded for tigecycline were 0.25 and 0.5–1 µg/ml, respectively. Minocycline was slightly less active than tigecycline, but the MIC_90_ values were also 0.5–1 µg/ml (data not shown). MIC_90_ values of levofloxacin were 2–4 µg/ml. Trimethoprim-sulfamethoxazole was not tested. 

### Gram-positive pathogens

#### Enterococcus spp.

The proportion of vancomycin-resistant (VRE) strains among the *E. faecium* isolates increased from 7.4% in 2006 to 31.6% in 2014 (Figure 1 (Fig. 1)), but remained very low among *E*. *faecalis* isolates (data not shown). Resistance rates for levofloxacin were very high (90.7% overall) for *E. faecium* and about 40% for *E. faecalis*. Further, resistance for ampicillin was low in *E. faecalis*, while 92.4% of *E. faecium* isolates were found to be resistant. In contrast, 99–100% of the *E. faecium* isolates, including VRE, remained susceptible to tigecycline and linezolid throughout the reporting period.

#### Staphylococcus aureus

The proportion of MRSA among *S. aureus* isolates (n=2,351) varied over the years, with an average rate of 20.4% (range 14.3% to 50.7%) with higher and lower MRSA rates in the first an second half of the reporting period, respectively (Figure 1 (Fig. 1)). Resistance to levofloxacin varied between 4.9% and 17.8% among MSSA isolates and between 73.3% and 98.1% among MRSA isolates. All *S. aureus* isolates tested were susceptible to tigecycline, linezolid and vancomycin. 

#### Streptococcus pneumoniae

The susceptibility pattern of *S. pneumoniae* remained largely unchanged during the observation period. Resistance rates for penicillin and ceftriaxone varied between 0% and 3.2% and those for levofloxacin between 0% and 2.7%, while the resistance rate for clarithromycin ranged from 8.5% to 22.5%, without a clear temporal trend. Resistance to linezolid was lacking and tigecyline showed MIC_50_ and MIC_90_ values of 0.015–0.03 µg/mL and 0.015–0.06 µg/mL, respectively.

## Discussion

Given the rapidly changing landscape of antimicrobial resistance, particularly in Gram-negative bacteria [29], long-term monitoring of the activity of available antibiotics against common and problematic pathogens involved in serious infections is of great importance in the management of infectious diseases. MRSA rates have been reported to decline in Germany in recent years [11], [30], [31], [32], while the rates of Gram-negative bacteria producing ESBLs and the rate of VRE remained either unchanged or increased over the past years [11], [31], [32], [33]. Efficient hygiene measures, antibiotic stewardship programmes and rational use of the available treatment options are crucial for the maintenance of the ability to control serious bacterial diseases, particularly in critically ill patients. Given the highly heterogenerous healthcare situations in Europan countries, regional longitudinal susceptibility data are key to enable adequate early action and policy adjustments if untoward trends in resistance emerge. The present study provides data from a larger and more diverse sample of isolates than previous surveys on the comparative susceptibility of tigecycline in Germany [12], [13], [14], [24].

The moderate increase in the prevalence of ESBL-producing *E. coli* and the stable proportion of ESBL-producing *K. pneumoniae* isolates found in the present study (Figure 1 (Fig. 1)) corresponds approximately with the ESBL rates reported by the Paul-Ehrlich-Gesellschaft für Chemotherapie (PEG) [31]. The low ESBL rate observed in *K. oxytoca* is explained by the low activity of the K1 beta-lactamase against the antibiotics cefotaxime and ceftazidime [34], which were used for ESBL screening. 

The rate of carbapenem-resistant *Acinetobacter* spp. showed a dramatic increase, reaching 33% in 2014, which is consistent with observations of other German surveillance studies for *A. baumannii* [31], [35].

Regarding Gram-positive pathogens, Gastmeier et al. [29] reported a strong increase in the proportion of VRE among nosocomial infections in Germany for the time period of 2007 to 2012. This observation corresponds to the trend towards higher VRE rates in the second half of the present study. 

MRSA rates varied considerably during the study period. The unusually high percentage of MRSA observed in 2009 is most likely due to random variation, given the low total number of isolates tested in that year. The downward trend of MRSA rates in the last three years, as also observed by other study groups in Germany, possibly reflects improved effects of infection control measures.

The clinical usefulness of tigecycline in the management of complicated infections, particularly cIAI and cSSTI, including those caused by pathogens with MDR has recently been confirmed in a large observational study programme performed in Germany [36], [37], [38]. These observations are supported by the results of the present multicentre in vitro study comprising more than 36,000 clinical isolates obtained from patients with community-acquired or nosocomial infections in Germany, confirming that eight years after its introduction into the German market, tigecycline invariably retains its high antimicrobial activity against a broad range of important Gram-negative and Gram-positive pathogens, including ESBL-producing Enterobacteriaceae, MRSA and VRE. The good activity of tigecycline found in the present study reassured the results of a German Tigecycline Evaluation and Surveillance Trial (G-TEST) performed between 2005 and 2009 [10], [11], [12]. Moreover, the German long-term findings for tigecycline from TEST are consistent with those published for Europe in general [39], and other individual European regions, particularly France [15], Italy [16], and Eastern European countries [17].

MIC_50_ and MIC_90_ values of 0.25 µg/mL and 0.5–1 µg/mL, respectively, assessed for *S. maltophilia* in the present study point to a potential usefulness of tigecycline in the mangement of infections caused by this opportunistic MDR pathogen of growing importance [40]. 

Tigecycline has been shown to be effective and well tolerated at higher than standard doses in critically ill patients infected with MDR bacteria [41] and in patients with hospital-acquired pneumonia [42]. Further studies investigating higher dosages of tigecycline in severly ill patients with difficult-to-treat infections appear warranted.

In conclusion, our findings indicate sustained activity of tigecycline against pathogens known to cause infections in severely ill patients. This is true for isolates susceptible to standard antibiotics as well as MDR bacteria like ESBL-producing Enterobacteriaceae, carbapenem-resistant *Acinetobacter* spp., VRE and MRSA, where choices of active drugs are generally limited or resistance rates worrisome.

## Notes

### Acknowledgements

The authors thank all investigators and staff of the participating laboratories and clinical centers for submitting bacterial isolates and the related data: University Hospital Aachen, Center for Microbiology and Infectiology Berlin, University Hospital Charité Berlin, Helios Hospital Berlin, Institute for Medical Laboratory Diagnostics Bochum, City Hospital Braunschweig, University Hospital Cologne, University Hospital Essen, University Hospital Frankfurt, University Hospital Freiburg, Medical Microbiology Laboratory Görlitz, University Hospital Halle, Regional Hospital Hannover, University Hospital Heidelberg, University Hospital Homburg/Saar, University Hospital Mannheim, University Hospital Kiel, University Hospital Leipzig, University Hospital Marburg, City Hospital Minden, Pettenkofer Institute Munich, University Hospital Münster, Antiinfectives Intelligence Rheinbach, University Hospital Tübingen, University Hospital Ulm.

Editorial support was provided by M. Fischer, Fischer BioMedical. This study was sponsored by Pfizer Inc. 

### Conflicts of interest

M.K. is a partner and CEO of Antiinfectives Intelligence GmbH, a research organisation providing services to pharmaceutical companies. 

B.K.-I. is an employee of Antiinfectives Intelligence GmbH.

C.P. is an employee of Pfizer Pharma GmbH.

H.S. has received grants or research support from the Bundesministerium für Bildung und Forschung (BMBF), Germany, the German Center for Infection Research (DZIF), Basilea, Novartis and Pfizer, has been a consultant for Astellas, AstraZeneca, Basilea, Cubist, Novartis, Pfizer, Tetraphase, and The Medicines Company, and has received payments for lectures from MSD, Novartis and Pfizer.

A.R. has been a consultant for Oxoid/Thermo, MSD, Novartis, InfectoPharm, BAG, Pfizer, Bayer, Siemens, Clinigen, Gilead and bestbion, and has received speakers honoraria from Pfizer.

K.B. has received grants or research support from from the Bundesministerium für Bildung und Forschung (BMBF), Germany, the German Center for Infection Research (DZIF), the Bundesministerium für Wirtschaft (BMWi), the European Territorial Cooperation (ETC)/INTERREG, Cepheid and Pfizer as well as lecture, travel and other fees from Cepheid, Cubist Pharmaceuticals, MSD Sharp & Dohme, Novartis Pharma, Oxoid, Pfizer and Siemens Healthcare Diagnostics.

## Figures and Tables

**Table 1:**
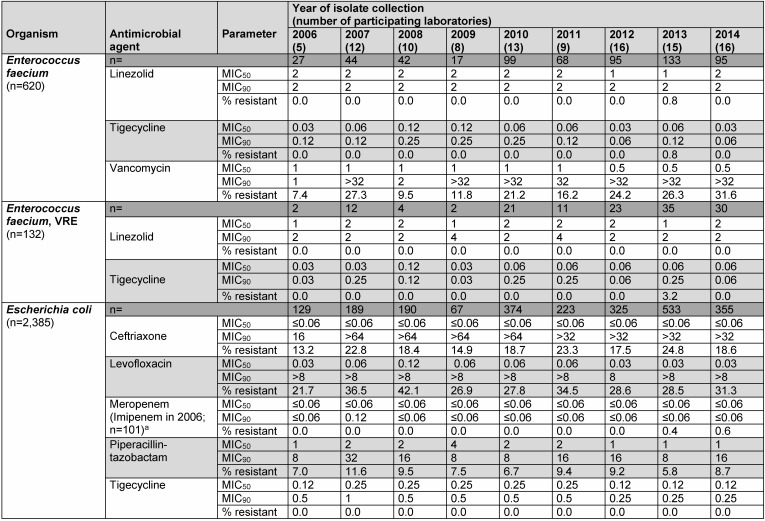
MIC_50/90_ values (µg/ml) and resistance rates (%) of bacterial isolates by species and year of collection in German TEST centers
